# A Strategy for Prompt Phase Transfer of Upconverting Nanoparticles Through Surface Oleate-Mediated Supramolecular Assembly of Amino-β-Cyclodextrin

**DOI:** 10.3389/fchem.2019.00161

**Published:** 2019-03-27

**Authors:** Xindong Wang, Guanying Chen

**Affiliations:** MIIT Key Laboratory of Critical Materials Technology for New Energy Conversion and Storage, Key Laboratory of Micro-systems and Micro-structures, School of Chemistry and Chemical Engineering, Harbin Institute of Technology, Ministry of Education, Harbin, China

**Keywords:** lanthanides, upconverting, phase transfer, amino-β-cyclodextrin, supramolecular self-assembly

## Abstract

Lanthanide-doped upconverting nanoparticles (UCNPs) are promising for applications as wide as biosensing, bioimaging, controlled drug release, and cancer therapy. These applications require surface engineering of as-prepared nanocrystals, commonly coated with hydrophobic ligand of oleic acid, to enable an aqueous dispersion. However, literature-reported approaches often require a long time and/or multiple step treatment, along with several fold upconversion luminescence (UCL) intensity decrease. Here, we describe a strategy allowing oleate-capped UCNPs to become water-soluble and open-modified, with almost undiminished UCL, through ultrasonication of minutes. The prompt phase transfer was enabled by oleate-mediated supramolecular self-assembly of amino modified β-cyclodextrin (amino-β-CD) onto UCNPs surface. We showed that this method is valid for a wide range of UCNPs with quite different sizes (6–400 nm), various dopant types (Er, Tm, and Ho), and hierarchical structures (core, core-shell). Importantly, the amino group of amino-β-CD on the surface of treated UCNPs provide possibilities to introduce entities for biotargeting or functionalization, as exemplified here, a carboxylic-containing near infrared dye (Cy 7.5) that sensitizes UCNPs to enhance their UCL by ~4,820 fold when excited at ~808 nm. The described method has implications for all types of oleate-capped inorganic nanocrystals, facilitating their myriad bioapplications.

## Introduction

Lanthanide-doped upconverting nanoparticles (UCNPs) are a new type of luminescent materials, which can produce visible or ultraviolet luminescence upon near infrared (NIR) excitation. They possess superior advantages over commonly seen luminescent materials (i.e., organic dyes, fluorescent proteins, and quantum dots), such as large anti-stoke shift, low imaging background, good colloidal stability, and deep tissue light activation (Chen et al., 2015; Wang et al., 2017). These merits empower them for uses in a comprehensive range of photonic and biophotonic applications, including solar cells, security encoding, and in particular, bio-imaging, biosensing, and light-activated cancer therapy. Most of these applications demand nanocrystal surface with critical engineered constituents, such as functional small molecule dyes, polymers, peptides, proteins, and nucleic acids, which, however, is generally unavailable from as-prepared UCNPs surface (Liu et al., 2011b; Wang et al., 2011; Chen et al., 2014, 2016).

The pristine ligands on the as-prepared UCNPs surface constitute the basic structure for surface engineering, which have an anchoring head group to coordinate to surface exposed metallic ions and an end group that points outside when dispersed. These ligands play a significant role in controlling the process of nanocrystal growth in solution as well as nanocrystal colloidal dispensability in solvents. Until now, commonly seen UCNPs with controlled and uniform sizes, shapes, crystal phases, are often synthesized with a high boiling point ligand of oleic acid (OA) (and sometimes together with oleylamine). The head group of OA is a carboxylic group, while the end group is a long alky chain, rendering them dispersible in non-polar organic solvents such as hexane. However, the hydrophobicity of the long alky chain prevents UCNPs for uses in aqueous media as well as for introduction of groups for biotargeting and functionalization. To solve this problem, a number of approaches have been developed and reported, such as ligand exchange (Wu et al., 2009; Shao et al., 2016; Wei et al., 2016; Lee et al., 2017), silica coating (Liu et al., 2013; Li et al., 2014b; Gnanasammandhan et al., 2016), oxidation of the C = C bond in the OA ligand (Chen et al., 2008; Zhou et al., 2009; Dai et al., 2012), assembly of amphiphilic polymers (Camli et al., 2010; Danhier et al., 2012; Zou et al., 2016), and other available methods (Salinas et al., 2015; Liu et al., 2017). However, most of these literature-reported methods typically either require a long time and multiple step treatment to render UCNPs water-soluble, or the obtained aqueous nanocrystals often lack open-modified groups, such as -NH_2_ and -COOH, for grafting needed functionalities (Li et al., 2014a, 2018). Moreover, these reported phase transfer processes are often in company with several fold UCL intensity decrease in UCNPs. It is still of importance to develop a simple and rapid approach to make OA-capped UCNPs hydrophilic and be open-modified for further functionalization.

Since the 1987 Nobel Prize in supramolecular chemistry, supramolecular recognition and self-assembly have been paid increased attentions in both scientific and technological developments (Yang et al., 2014; Mattia and Otto, 2015; Lehn, 2017). Cyclodextrin (CD), a water-soluble cyclic supramolecule, has a rigid well-defined ring structure and is able to firmly bind to some particular low-molecular-weight compounds, endowing these compounds new physiochemical properties (Harada, 2001; Descalzo et al., 2006). Indeed, recent results show that CD and its derivatives are able to form supramolecular complexes with OA molecules, providing possibilities for nanocrystal surface treatment to control their dispersibility properties (Wang et al., 2003; Liu et al., 2011a; Omer et al., 2011). Compared with commonly used surface treatment methods, the method of CD modification is simple and prompt (only needs ultrasonication for several minutes), leaving the pristine ligands anchored on UCNPs surface intact. As a result, this surface treatment method could avoid the formation of surface defects for energy trapping that typically leads to the decrease of UCL quantum yield.

In this work, we synthesized amino modified β-cyclodextrin (amino-β-CD) and then applied it to OA-capped UCNPs of varying type (different sizes, various types of lanthanide dopants, and core-shell structure), enabling them water-soluble and open-modified for functionalization. The interaction between the host molecule of amino-β-CD and the guest surface molecule of OA allowed a prompt, stable, and straightforward self-assembly of amino-β-CD onto the surface of UCNPs through ultrasonication of 2–4 min. Experimental results showed that the morphology, crystallographic phase, and UCL intensity of UCNPs are retained to a maximum. Importantly, the amino groups contained in the surface CD molecules allow to introduce a carboxylic-containing dye (Cy 7.5) onto the nanocrystal surface that can sensitize the nanocrystal to enhance its UCL by ~4,820 fold when excited at ~808 nm.

## Materials and Methods

### Reagents And Apparatus

Oleic acid (OA) and 1-octadecene (ODE) were purchased from Sigma Aldrich (USA). Rare earth chloride hexahydrate (YCl_3_·6H_2_O 99.9%, YbCl_3_·6H_2_O 99.9%, ErCl_3_·6H_2_O 99.9%, TmCl_3_·6H_2_O 99.9%, and HoCl_3_·6H_2_O 99.9%), rare earth nitrates (Y(NO_3_)_3_·6H_2_O 99.9%, Yb(NO_3_)_3_·5H_2_O 99.9%, and Er(NO_3_)_3_·5H_2_O 99.9%), sodium oleate, ammonium hydroxide, β-CD, epoxy chloropropane, neutral alumina (100–200 mesh), and sodium fluoride (NaF) were from Aladdin (Shanghai, China). Hexane, methanol, ethanol, sodium hydroxide (NaOH), and ammonium fluoride (NH_4_F) were purchased from Xilong Scientific Co., Ltd (Guangdong, China). Yttrium oleate was prepared according to a method in a literature (Park et al., 2004). All the reagents were used without further purification.

The transmission electron microscope (TEM) images were acquired with a 100 CX II TEM microscope (JEM, Japan). Fourier transform infrared (FTIR) spectra were recorded on a Nicolet AVATAR360 FTIR spectrometer (Thermo Nicolet Corporation, USA). Luminescence spectra were obtained at a FLAME-T-VIS-NIR Spectrometer Assembly (Ocean Optics, USA) with an excitation from a ~980 or 808 nm diode laser (CNI, Changchun). The integration time for the spectrometer was set to 600 ms, while the power of 980 and 808 nm laser was set to 400 and 300 mW, respectively, for all pertinent optical experiments. ^1^H nuclear magnetic resonance (NMR) spectra were recorded on Bruker Avance 500 (500 MHz, USA). Luminescence lifetimes were recorded on the FLS 980 transient fluorescence spectrometer (Edinburgh Instruments, England).

### Preparation of NaYF_4_: Yb, Er UCNPs

#### Preparation of Small Size (6 nm) NaYF_4_: 30%Yb, 2%Er UCNPs

Small size NaYF_4_: 30%Yb, 2%Er UCNPs (6 nm) were synthesized according to a previous procedure with modifications (Rinkel et al., 2016). Typically, 1.59 g of rare earth oleates (Y, Yb and Er oleates with a molar ratio of 0.68: 0.3: 0.02) and 4.06 g of sodium oleate were firstly combined with 20 mL OA and 20 mL octadecene in a 100 mL three neck flask. The mixture was degassed for 1 h at 100°C under Ar atmosphere and vigorous stirring. Then, 0.68 g of NH_4_F was added to the solution, which maintained at 100°C for 30 min with Ar atmosphere protection. Subsequently, the reaction mixture containing sodium oleate, rare-earth oleates and NH_4_F in a molar ratio of 8:1:11 was heated to 300°C with a heating rate of 16°C/min, and then kept at this temperature for 30 min. After cooling to room temperature, excess ethanol was added to the solution to precipitate the nanocrystals, followed by centrifugation at 10,000 rpm/min for 5 min. The collected precipitate was redispersed in a small amount of hexane, and then precipitated again with ethanol. Finally, the resultant UCNPs were collected with centrifugation and redispersed in 10 mL hexane.

#### Preparation of Middle Size (42 nm) NaYF_4_: 30%Yb, 2%Er UCNPs

Middle size NaYF_4_: 30%Yb, 2%Er particles (42 nm) were synthesized as follows: 0.2063 g YCl_3_.6H_2_O, 0.1162 g YbCl_3_.6H_2_O, and 0.0076 g ErCl_3_.6H_2_O (Y, Yb and Er chlorides in a molar ratio of 0.68: 0.3: 0.02) were mixed with 9 mL OA and 15 mL octadecene in a 100 mL three neck flask. The slurry mixture was heated to 150°C and kept for 30 min to form a transparent solution under Ar flow protection. Then the mixture was cooled down to 50°C, followed by addition of 10 mL methanol containing 0.1482 g NH_4_F and 0.1000 g NaOH, and then stirred for 30 min. Subsequently, the mixture was heated to 80°C and kept at this temperature for 30 min in order to evaporate methanol. Then the mixture was heated to 300°C with a rate of 15°C/min under Ar flow, and maintained at 300°C for 60 min. After cooling to room temperature, excess ethanol was added to the solution to precipitate the nanocrystals, followed by centrifugation at 6,000 rpm/min for 5 min. The precipitate was collected and redispersed in a small amount of hexane, and then precipitated again with ethanol. Finally, the particles were separated by centrifugation and redispersed in 10 mL hexane.

#### Preparation of Large Size (400 nm) NaYF_4_: 30%Yb, 2%Er UCNPs

Firstly, 1.0846 g NaOH was mixed with 4 mL H_2_O, 12 mL OA, and 6 mL ethanol, and stirred for 30 min. Then, a certain amount of rare earth nitrates (Y, Yb, and Er nitrates in a molar ratio of 0.68: 0.3: 0.02) were added into the mixture and stirred for another 10 min. Subsequently, 4 mL NaF (1.25 M) solution was added and stirred for 30 min. Finally, the solution was transferred to a stainless Teflon autoclave (50 mL), sealed, and heated at 180°C for 24 h. After cooling to room temperature, excess ethanol was added to the solution to precipitate UCNPs, followed by centrifugation at 6,000 rpm/min for 5 min. The precipitate was then redispersed in a small amount of hexane, and precipitated again with ethanol. Finally, the resultant particles were separated by centrifugation and redispersed in 10 mL hexane.

#### Preparation of Middle Size (42 nm) NaYF_4_: Yb, X (X = Er, Tm, and Ho) UCNPs

The synthesis procedures are similar with the one of middle size NaYF_4_: Yb, Er particle. A total amount of 1 mmol rare earth chloride hexahydrate (Y+Yb+X, X = Er, Tm, and Ho) were mixed with 9 mL OA and 15 mL octadecene. The slurry mixture was heated to 150°C and kept for 30 min to form a transparent solution under Ar flow. Then the mixture was cooled down to 50°C, followed by addition of 10 mL methanol containing 0.1482 g NH_4_F and 0.1000 g NaOH, and stirred for 30 min. The mixture was then heated to 80°C and kept for 30 min in order to evaporate methanol. Subsequently, the solution was heated to 300°C with a rate of 15°C/min under Ar flow, and maintained at 300°C for 60 min. After naturally cooling to room temperature, excess ethanol was added to precipitate the nanocrystals followed by centrifugation at 6,000 rpm/min for 5 min. The precipitate was then redispersed in a small amount of hexane and precipitated again with ethanol. Finally, the particles were separated by centrifugation and redispersed in 10 mL hexane.

#### Preparation of Middle Size Core-Shell (64 nm) NaYF_4_: Yb, Er@NaYF_4_ UCNPs

The middle size core particles (42 nm) of NaYF_4_: Yb, Er were synthesized using the above-mentioned procedure. The coating procedure of the inert shell layer of NaYF_4_ was described as follows. First, 0.5 mmol of yttrium hexhydrate chloride was mixed with 9 mL OA and 15 mL octadecene in a 100 mL three necked flask. The slurry mixture was then heated to 150°C and kept at this temperature for 30 min to form a transparent solution under Ar atmosphere protection. Subsequently, the mixture was cooled down to 50°C, followed by addition of 10 mL methanol containing 0.0741 g NH_4_F and 0.0500 g NaOH, and 10 mL hexane containing the 42 nm core NaYF_4_:Yb, Er particles. After magnetic stirring at this temperature for 30 min, the mixture was heated to 80°C and kept at this temperature for 30 min to evaporate methanol. The mixture was then heated to 300°C with a rate of 15°C/min under Ar atmosphere protection, and maintained at this temperature for 60 min. After cooling to room temperature, excess ethanol was added to precipitate the nanocrystals from the mixture, followed by centrifugation at 6,000 rpm/min for 5 min. The precipitate was redispersed in a small amount of hexane and then precipitated with addition of excessive ethanol. Finally, the particles were collected by centrifugation and redispersed in 10 mL hexane.

### Preparation Of Amino-β-CD

**Figure 1:**
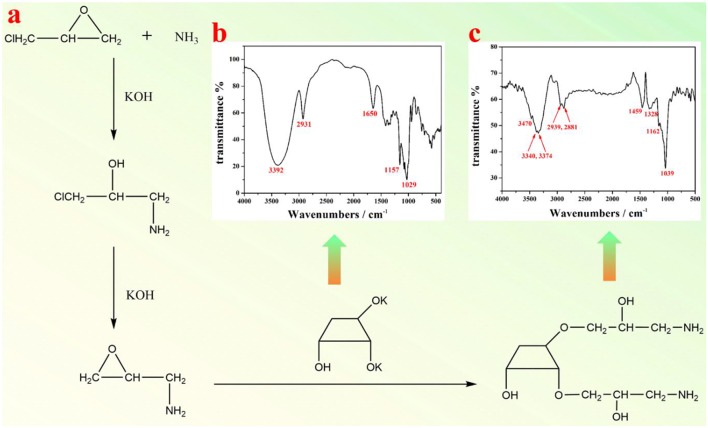
**(a)** A schematic illustration of synthesis of amino-β-CD; FTIR spectra of **(b)** β-CD, and **(c)** amino-β-CD.

As shown in Figure 1a, the epoxy chloropropane was used to crosslink the amino group and the β-CD in a dilute alkali solution. The procedure to prepare amino modified β-CD is described as follows. Firstly, 8.1 g β-CD and 6.7 g KOH were added into 70 mL H_2_O, which was kept stirring until β-CD was completely dissolved. The resultant mixture was then heated to 50°C, to which 3.4 g ammonium hydroxide and 10.2 g epoxy chloropropane were added in order. Subsequently, the mixture solution was heated to 60°C and maintained at this temperature for 1 h. When completing the reaction, the pH of the solution was first adjusted to 5–6, and then 150 mL ethanol was poured into the solution at room temperature. The final product was purified via neutral alumina column, using an eluent of 60% ethanol. The resultant mixture was concentrated to 30 mL by evaporating redundant ethanol, followed by adding a large amount of absolute methanol and placed overnight. Finally, the amino-β-CD product was filtrated and dried in vacuum.

FTIR spectra of pristine β-CD (Figure 1b) and amino functionalized β-CD (Figure 1c) were both measured. As shown in Figure 1b, the bands at 2,931 cm^−1^ and 1,334–1,440 cm^−1^ represent the stretching and bending vibration of alkane (C-H). The bands at 3,392 and 1,650 cm^−1^ represent the stretching and bending vibration of O-H, and the bands at 1,029 and 1,157 cm^−1^ are both from the stretching vibration of C-O. After amino group modification, the vibration bands of O-H and C-H are both changed (move or split, Figure 1c), which are possibly due to the influence of linked amino alkane. Furthermore, the bending vibrations of O-H at 1,650 cm^−1^ and the stretching vibration of C-O at 1,157 cm^−1^ almost disappear, since the -OH group has been replaced by the amino alkane group. The appearance of the stretching vibration of the N-H group at 3,470 cm^−1^ clearly indicates the presence of amino group, and thus the successful preparation of amino-β-CD. The NMR spectrum of amino functionalized β-CD was shown in Figure S1. ^1^H NMR of amino-β-CD (500 MHz, DMSO-*d*_6_) δ 8.84 (s, 4H), 7.49 (s, 1H), 7.39 (s, 1H), 7.28 (s, 1H), 5.78–5.71 (m, 4H), 5.59 (s, 2H), 5.03 (s, 6H), 4.84 (s, 3H), 4.58 (s, 6H), 4.03 (s, 2H), 3.99–3.94 (m, 1H), 3.87 (s, 2H), 3.69 (s, 15H), 3.63 (s, 12H), 3.56 (s, 3H), 3.36 (ddt, *J* = 31.8, 15.0, 6.9 Hz, 14H), 3.23–3.16 (m, 21H), 3.00 (s, 1H), 2.87 (s, 2H).

### Surface Modification of OA-Capped UCNPs With Amino-β-CD

**Figure 2:**
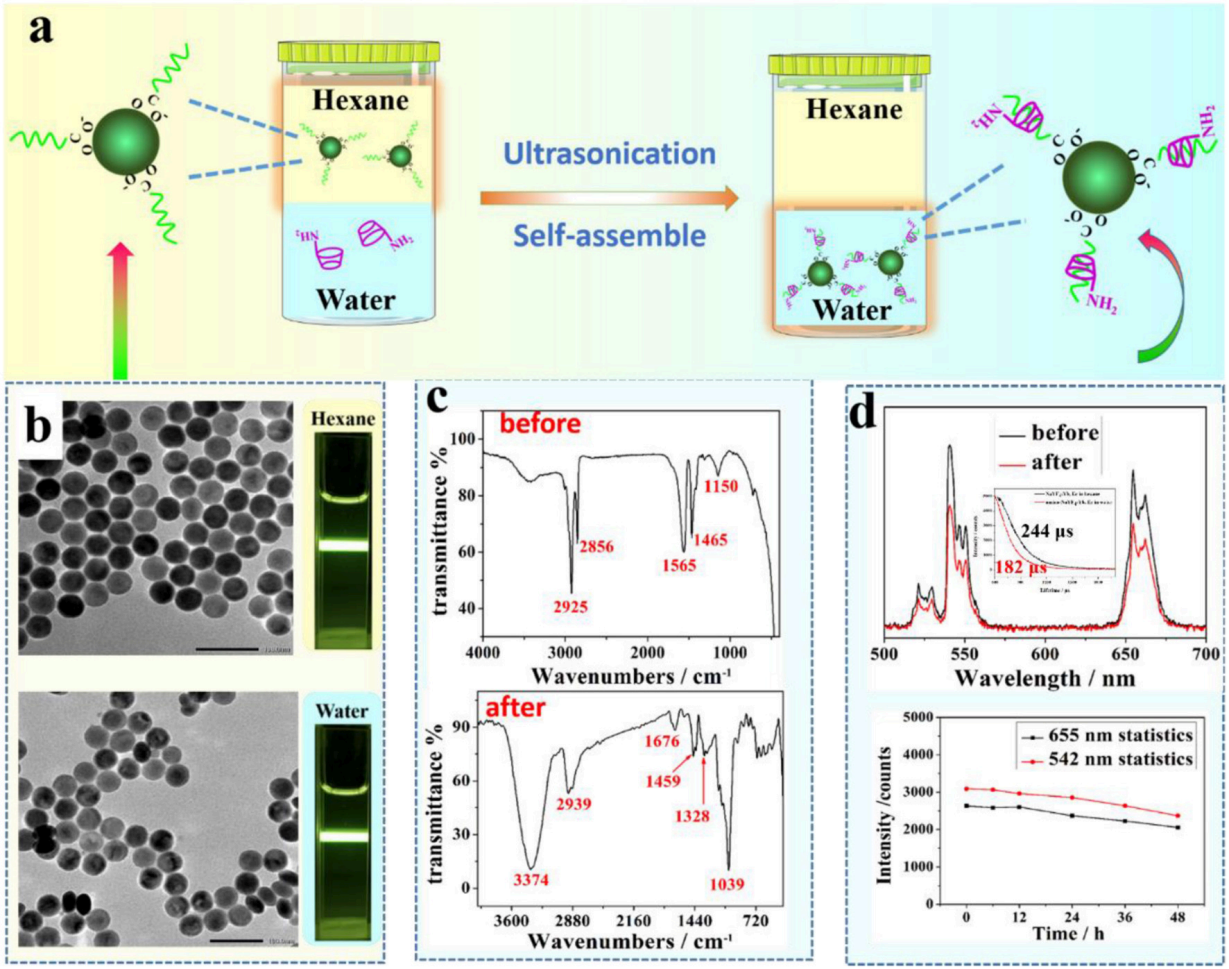
**(a)** A schematic illustration of the phase transfer process of UCNPs from hexane to water; **(b)** TEM images of NaYF_4_:Yb, Er UCNPs before (upper figure) and after (bottom figure) surface treatment, **(c)** FTIR spectra of UCNPs before (upper figure) and after (bottom figure) surface treatment with amino-β-CD; **(d)** A comparison of UCL spectra of NaYF_4_:Yb, Er UCNPs before and after surface treatment (upper figure); The inert is the relevant lifetime changes. an evaluation of water stability of amino-β-CD modified UCNPs through monitoring UCL peak change at 655 and 542 nm over a period of time (bottom figure).

Surface treatment of OA-capped UCNPs was realized through an OA-targeting supermolecular self-assembly of amino-β-CD on to the nanoparticle surface (consult Figure 2a). In a typical procedure, a certain amount (1 mL) of as-prepared UCNPs in hexane (concentration of 10 mg/mL) were dispersed in 10 mL water solution containing amino-β-CD (10 mg/mL) and 0.06 M hydrochloric acid. The mixture was ultrasonically treated for 2–4 min at room temperature. Note that, longer ultrasound time is needed for smaller sized particles (6 nm). Finally, the resultant mixture was centrifugally separated to collect the product, which was then washed three more times with 10 mL deionized water, and finally stored in 5 mL deionized water.

### Attachment of Cy 7.5 to Amino-β-CD Modified NaYF_4_: Yb, Er UCNPs

The attachment of near infrared Cy 7.5 dye to the surface of amino β-CD modified NaYF_4_: Yb, Er UCNPs was realized through direct formation of a covalent amide bond between the carboxyl group of Cy 7.5 dye and the amino moiety of amino β-CD, with presence of cross-linking reagents (EDC/NHS) (consult **Figure 5A**). Specifically, 100 μL of Cy 7.5 dye water solution (2 mg/mL) was added into 1 mL water solution containing EDC (400 mM) and NHS (100 mM), which was freshly prepared before the use for activation of the carboxyl group for 2 h. Then, 2 mL of amino-β-CD modified UCNPs (10 mg/mL) water solution were added to the activated Cy 7.5 solution, and stirred for 4 h at room temperature. Finally, the resultant mixture was centrifugally separated to collect the product, followed by washing three times with 10 mL dimethyl formamide (DMF), and then stored in 5 mL DMF for measurements.

## Results and Discussion

Figure 2 shows the result of surface treatment of middle size NaYF_4_:Yb, Er UCNPs (42 nm) with amino-β-CD through ultrasonication of two min. An intercalation of the long alky chain of OA molecules with the rigid ring of β-CD allows a stable attachment of amino-β-CD molecules to the nanocrystal surface (Figure 2a). The hydrophilicity of amino-β-CD, therefore, imparts a phase transfer of hydrophobic OA-capped UCNPs from organic phase (hexane) in the upper layer to aqueous phase at the bottom. A successful phase transfer can be indicated by an observation of strong UCL from surface-treated UCNPs in an aqueous dispersion (Figure 2b). To further demonstrate the successful phase transfer, we acquired FTIR spectra of UCNPs before and after amino-β-CD surface treatment (Figure 2c). Before surface treatment, the bands at 2,856/2,925 cm^−1^ and 1,465 cm^−1^ are from the stretching and bending vibration of alkane (C-H), while the bands at 1,150 and 1,565 cm^−1^ represent the stretching vibration of C-O and C = O groups, arising from the OA ligand molecule on the surface. After surface modification, the vibration bands of OA at both 1,150 and 1,565 cm^−1^ nearly disappear, being attributed to the host-guest intercalation of the amino-β-CD and the OA molecule. In addition, the characteristic vibration bands at 1,039, 1,328, 1,459, 2,939, and 3,374 cm^−1^ of amino-β-CD molecule (Figure 1), emerges after surface treatment, which indicates the successful attachment of amino-β-CD molecules onto the surface of OA-capped UCNPs.

The amino-β-CD surface treatment procedure does not produce overt effects on UCNPs size and morphology, as the NaYF_4_:Yb, Er UCNPs, before and after treatment, are both shown to be spherical with a narrow size distribution of about 42 nm (Figure 2b). In addition, UCL spectra of the middle size UCNPs before (dispersed in hexane) and after (dispersed in water) amino-β-CD treatment were acquired and shown in Figure 2d. The UCL peaks at 525/542 and 655 nm, located in the visible spectral region, correspond to the ^2^H_11/2_/^4^S_3/2_ → ^4^I_15/2_, and ^4^F_9/2_ → ^4^I_15/2_ transitions of Er^3+^ ions, respectively, in good agreement with previously reported results (Chen et al., 2014). In addition, after phase transfer to water, the lifetime of NaYF_4_:Yb, Er UCNPs slightly decreases from 244 to 182 μs, implying that the integrity of prestine surface was largely retained (Figure 2d). The UCL intensity remain almost undiminished after the phase conversion from hexane to water, showing a slight decrease of 1.5 fold. This conclusion can also be suggested from a direct comparison of UCL photographic images in both hexane and water (Figure 2b). To investigate the stability of amino-β-CD modified UCNPs, room temperature UCL spectra of these surface-treated UCNPs were measured at different time points (0, 6, 12, 24, 36, and 48 h) over a period of 2 days. As shown in the bottom of Figure 2d, the luminescence intensities of Er at 542 and 655 nm were merely slightly decreased with an increase of time, and retained about 90% of UCL intensity for up to 2 days, demonstrating the good stability of amino-β-CD surface-treated UCNPs. The amino-CD modified nanoparticles also showed good photostability using 400 mW 980 nm laser illuminated for 30 min (Figure S2), and great chemical stability when aged in water for 12 h (Figure S3).

**Figure 3:**
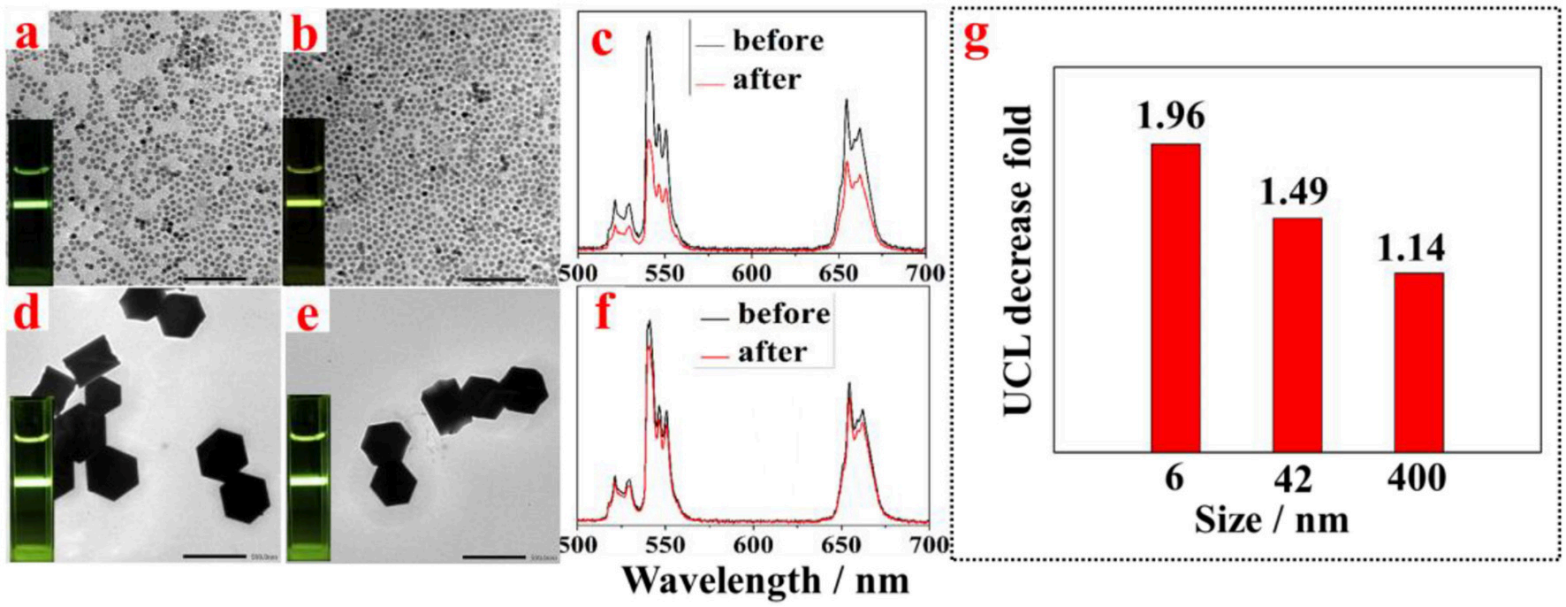
TEM images of NaYF_4_:Yb, Er UCNPs with small size before **(a)** and after **(b)** surface treatment, and with large size before **(d)** and after **(e)** surface treatment; the corresponding UCL spectra from NaYF_4_:Yb, Er UCNPs with **(c)** small size and **(f)** before and after surface treatment; **(g)** The observed UCL decrease fold vs. the size of NaYF_4_:Yb, Er UCNPs after surface treatment with amino-β-CD.

To test whether the described approach can be valid for small and large OA-capped UCNPs, we synthesized UCNPs of small size (6 nm) and large size (400 nm) and implemented the phase transfer procedure. TEM images of the NaYF_4_:Yb, Er UCNPs of small and large size UCNPs before and after amino-β-CD modification are correspondingly shown in Figures 3a,b,d,e, respectively. As expected, no overt changes of morphology and size were observed. UCNPs of both small and large sizes were successfully transferred into aqueous phase, displaying intense UCL in water (small size, the inset of Figure 3b; large size, the inset of Figure 3e). The UCL intensity from UCNPs of both sizes (small size, Figure 3c; large size, Figure 3f) were almost retained. This can also be seen from a comparison of UCL photographic images before and after phase transfer (small size, the inset of Figure 3a vs. Figure 3b; large size, the inset of Figure 3d vs. Figure 3e). Note that the fold of surface treatment induced UCL decrease diminishes with an increase of the size of UCNPs, from ~2 fold for 6 nm UCNPs to ~1.1 fold for 400 nm UCNPs (Figure 3g). This observation possibly stems from the decreased surface to volume ratio with an increase of particle size. It is known that the large surface to volume ratio can produce pronounced UCL quenching effect, as a large number of rare earth ions will be exposed to surrounding quenching centers (surface defects, high energy vibrations from ligands and solvents) (Chen et al., 2014). As a result, after phase transfer, smaller size UCNPs with larger surface to volume ratio are more prone to be quenched by water molecules from solvent, thus resulting in higher UCL decrease. However, it should be noted that the observed maximum UCL decrease is merely 2 fold for small size UCNPs (6 nm). This result indicates that the intercalation of amino-β-CD to the surface of OA-capped UCNPs is able to retain the integrity of pristine particles, without creating noticeable surface defects for substantial UCL quenching that were commonly seen in literature- reported phase transfer methods.

**Figure 4:**
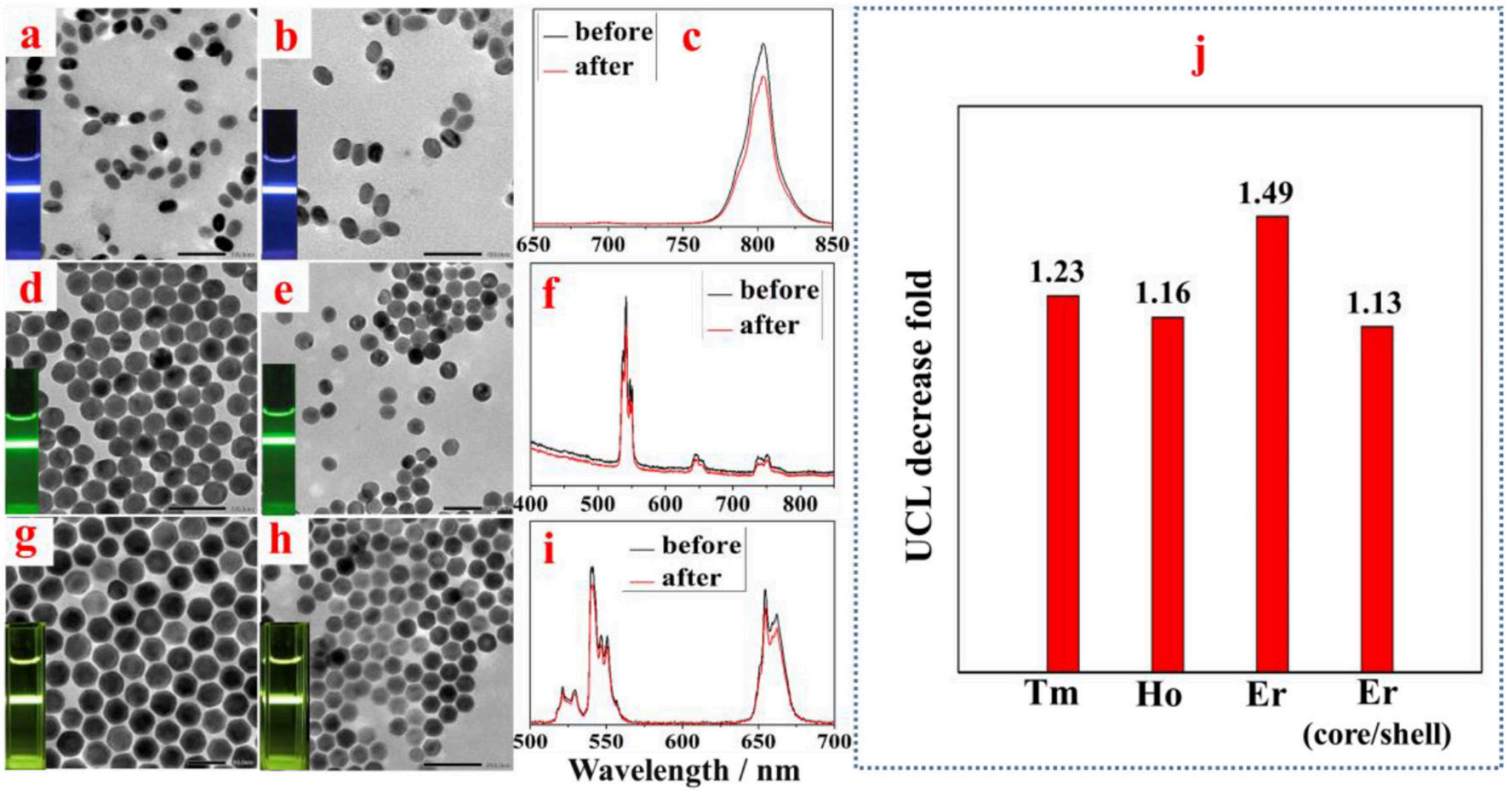
TEM images of middle size NaYF_4_:Yb,Tm **(a,b)**, NaYF_4_:Yb, Ho **(d,e)**, and core-shell NaYF_4_:Yb, Er@NaYF_4_ UCNPs **(g,h)** before and after surface treatment. The corresponding UCL spectra from NaYF_4_:Yb,Tm **(c)**, NaYF_4_:Yb, Ho **(f)**, and core-shell NaYF_4_:Yb, Er@NaYF_4_ UCNPs **(i)**, respectively. The column graph data in **(j)** depicts a comparison of surface treatment induced UCL decrease fold for UCNPs doped with different activators (Tm, Ho, and Er) or with core/shell structure.

To investigate the effect of amino-β-CD treatment on the UCL of other types of UCNPs, we prepared a set of middle size (42 nm) UCNPs with different emission bands and with a core/shell structure (core 42 nm, core-shell 64 nm). As shown in Figure 4, TEM images of NaYF_4_:Yb, X (X = Tm, or Ho) UCNPs (42 nm) and NaYF_4_:Yb, Er@NaYF_4_ core-shell UCNPs (64 nm) before and after amino-β-CD modification present no identifiable changes on the morphologies and sizes, as expected. In addition, no spectral and relative intensities changes were observed for both NaYF_4_:Yb, X (X = Tm, or Ho) UCNPs and NaYF_4_:Yb, Er@NaYF_4_ core-shell UCNPs after surface modification with amino-β-CD. The UCL intensities show a slight decrease after the conversion from hexane to water, which can be ascribed to the quenching effect induced by energetic hydroxyl (-OH) group of water molecules. However, it should be noted that the UCL quenching fold for middle size (42 nm) NaYF_4_:Yb, Tm and NaYF_4_:Yb, Ho are both about 1.2, a little smaller than the 1.5 fold for middle size (42 nm) NaYF_4_:Yb, Er, presenting negligible quenching effect (Figure 4j). Importantly, the UCL quenching fold for the NaYF_4_:Yb, Er@NaYF_4_ core-shell UCNPs is just 1.1 fold, displaying almost identical UCL intensity before and after amino-β-CD treatment. This indicates that the aqueous environment produces no quenching effect for UCL from the core/shell structure UCNPs, which can possibly be due to the spatial isolation of the core from the surrounding environment.

**Figure 5:**
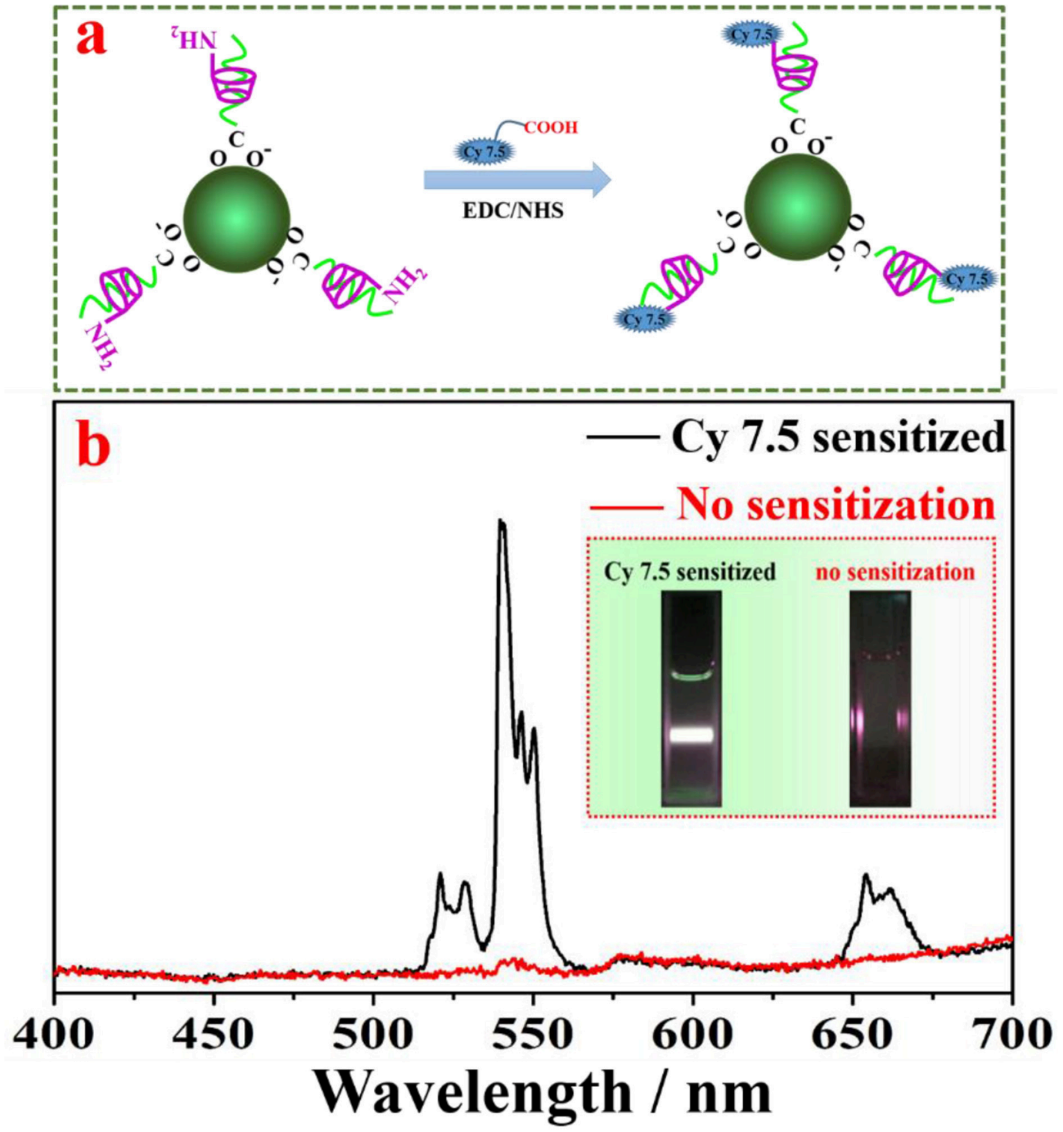
**(a)** A schematic illustration of grafting Cy 7.5 dyes to the surface of UCNPs; **(b)** UCL spectra of NaYF_4_:Yb, Er UCNPs with and without Cy 7.5 dyes on the nanocrystal surface, showing UCL enhancement of ~4,820 fold due to the antenna effect of the grafted dye.

The amino group on the surface of lanthanide-doped nanocrystals provide numerous open-modified opportunities for further functionalizations through a covalent linkage of functional molecules, such as dye, protein, and ribonucleic acid. As a proof-of-concept experiment, a type of carboxyl group functionalized near infrared dye (Cy 7.5) was linked onto the surface of NaYF_4_:Yb, Er UCNPs through formation of an amide bond with the amino group contained in amino-β-CD on the nanocrystal surface (Figure 5a). It has been shown that organic dye sensitization is promising to solve the weak and narrow absorption problem of lanthanide-doped UCNPs, as the absorption of an organic dye is three orders of magnitude higher than that of a lanthanide ion. After light absorption, efficient nonradative energy transfer from organic dyes to the ytterbiurm (Yb) ions on the nanocrystal surface can empower an efficient photon upconversion through well-established Yb-X (X = Er, Ho, Tm) interactions, here X = Er (Chen et al., 2015; Wang et al., 2017). The β-CD modified NaYF_4_:Yb, Er UCNPs without Cy 7.5 dyes served as a control sample. As shown in Figure 5b, under an excitation at 808 nm, the Cy 7.5 linked-NaYF_4_:Yb, Er UCNPs exhibit intense UCL with characteristic peaks of Er at 542 and 655 nm, in marked contrast to none UCL from the control sample. The sensitization effect can take place through either covalently linked dyes or non-covalently linked dyes. We estimated about 80% sensitization enhancement originates from the covalent linkage of Cy 7.5 dyes (Figure S4). In addition, we calculated that the integrated UCL from Cy 7.5 dye-modified UCNPs in the spectral range of 400–700 nm was about ~4,820 fold higher than that of UCL from the control sample. This significant UCL enhancement can be clearly discerned from the photographic UCL images (the inset of Figure 5b), which unambiguously originated from the sensitization of the NaYF_4_:Yb, Er UCNPs by the linked Cy 7.5 dye on the UCNPs surface.

## Conclusions

In summary, we have developed a simple approach for prompt conversion of hydrophobic lanthanide-doped UCNPs, commonly capped with OA ligand, to be water-soluble and open-modified for functionalization, based on OA-targeting supramolecular self-assembly of amino-β-CD. This method was shown to be valid for UCNPs with a broad spectrum of sizes (6–400 nm), a set of rare earth dopants (Yb/Er, Yb/Ho, and Yb/Tm), as well as core-shell structure through ultrasonication of 2–4 min. Importantly, UCL intensities from surface treated UCNPs were almost identical to their parent OA-capped UCNPs. Moreover, these amino-β-CD modified UCNPs were found to be stable over 48 h without overt diminishment of their UCL intensities. The amino group on the surface of resultant amino-β-CD modified UCNPs creates opportunities to graft other functionalities, as exemplified here, by a covalent linkage of the carboxylic-containing dye (Cy 7.5) to the surface, which sensitizes 42 nm NaYF_4_:Yb/Er UCNPs, enhancing their UCL by ~4,820 fold (when excited at 808 nm). The described approach here holds great promise for surface treatment of other kinds of OA or OA-analogs capped inorganic nanocrystals, fostering their applications in nanomedicine and theranostics.

## Author Contributions

XW and GC conceived the idea and designed the investigation. XW contributed to the synthesis and characterization of the materials. XW and GC analyzed the data and wrote the manuscript. All authors approved the submitted version.

### Conflict of Interest Statement

The authors declare that the research was conducted in the absence of any commercial or financial relationships that could be construed as a potential conflict of interest.
