# Supplementary material for: A Strategy for Prompt Phase Transfer of Upconverting Nanoparticles Through Surface Oleate-Mediated Supramolecular Assembly of Amino-β-Cyclodextrin
Source: Front Chem. 2019 Mar 27;7:161. doi: 10.3389/fchem.2019.00161 (PMC6445860; doi:10.3389/fchem.2019.00161)
Supplement: Supplementary file 1 [file Data_Sheet_1.docx]

**Supplementary Information for**

**A strategy for prompt phase transfer of upconverting nanoparticles through surface oleate-mediated supramolecular assembly of amino-β-cyclodextrin**

Xindong Wang and Guanying Chen*

MIIT Key Laboratory of Critical Materials Technology for New Energy Conversion and Storage, School of Chemistry and Chemical Engineering & Key Laboratory of Micro-systems and Micro-structures, Ministry of Education, Harbin Institute of Technology, Harbin, People’s Republic of China.

* Correspondence: [chenguanying@hit.edu.cn](mailto:chenguanying@hit.edu.cn)


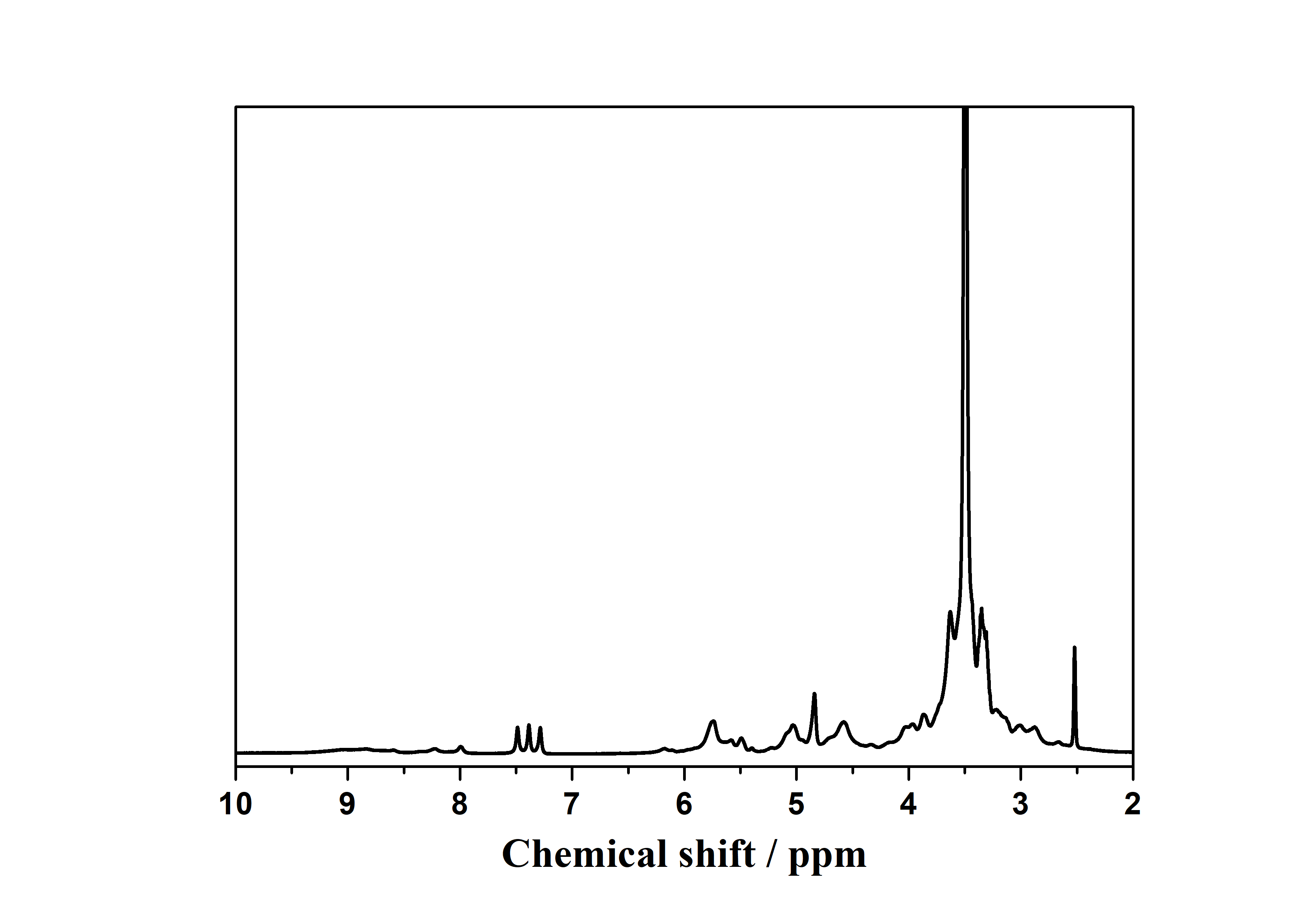


**Figure S1.** ^1^HNMR spectra of amino-β-CD in DMSO-*d*_6._





**Figure S2.** Photostability of amino-CD modified NaYF_4_:Yb, Er in water (laser power of 400 mW, for 30 min). I and I_0_ refer to the detected intensity and original intensity, respectively. The constant ratio indicates a good photostability of these amino-CD modified nanoparticles.


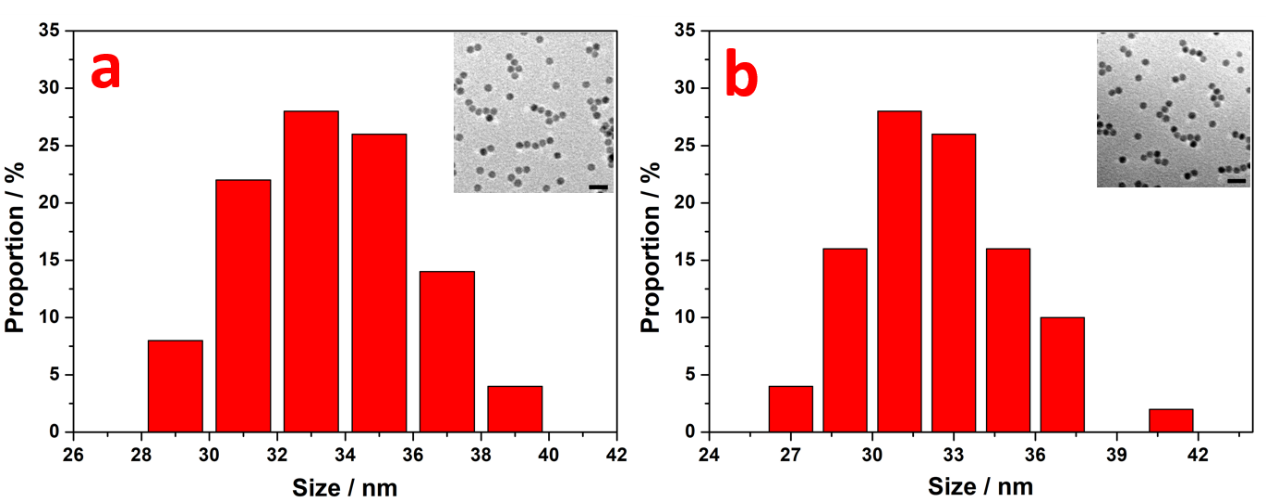


**Figure S3.** Size distributions and TEM images of (a) as-synthesized amino-CD modified NaYF_4_:Yb, Er and (b) amino-CD modified UCNPs aged in water for 12 h. The unaltered morphology and size distribution indicate a good chemical stability of these amino-CD modified nanoparticles.


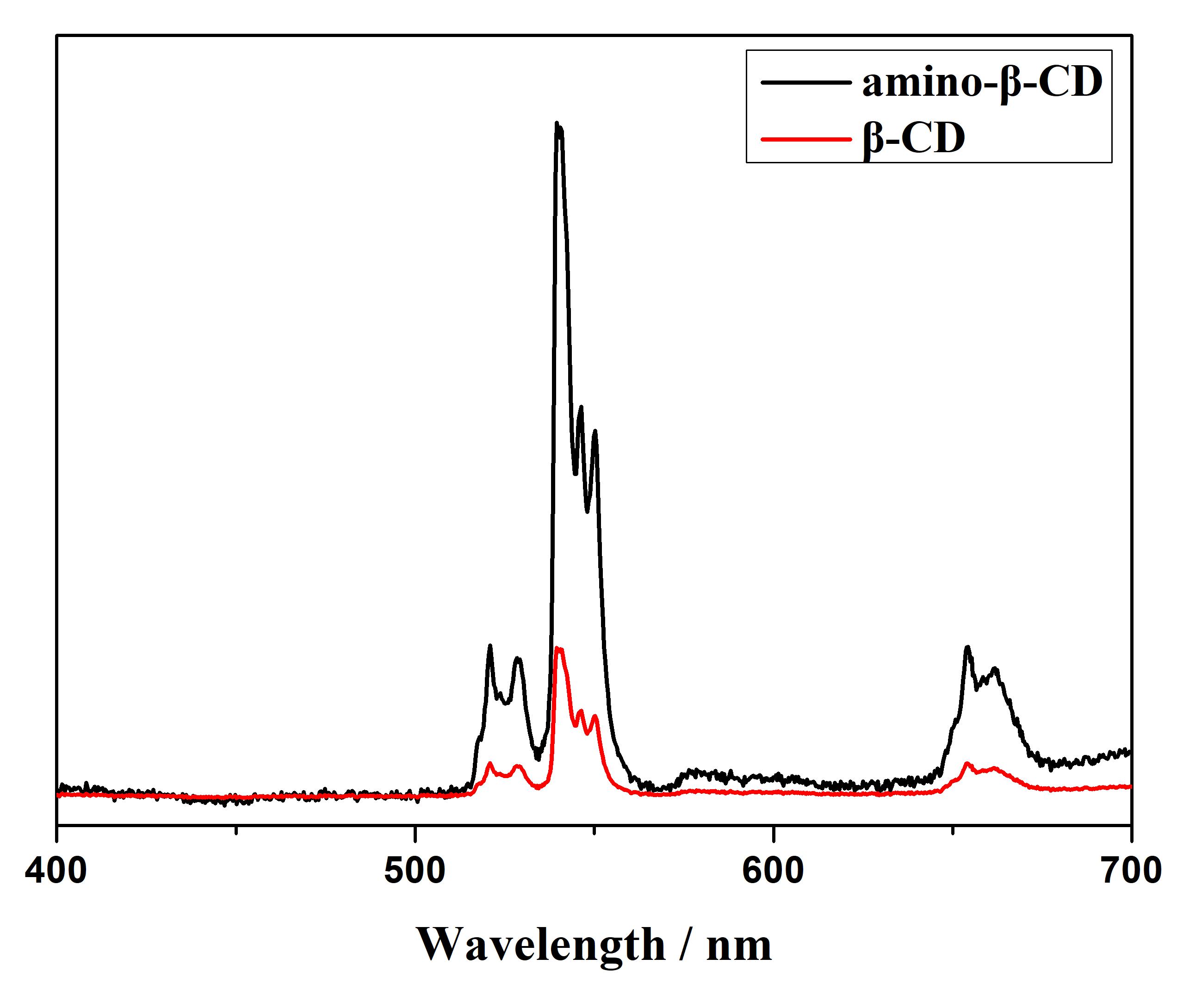


**Figure S4.** UCL spectra of amino-CD modified UCNPs and CD-modified UCNPs sensitized with Cy 7.5 dyes. It can be concluded that covalent linkage of Cy 7.5 dye to amino-CD are about five times more efficient than the noncovalent of Cy 7.5 dye, implying that the means of covalent linkage contributes to about 80% of the observed dye sensitization enhancement effect.
